# Intersections of Compassion, Science, and Spiritual Care in Global Health for Public Health Benefits

**DOI:** 10.1007/s10943-024-02145-x

**Published:** 2024-10-01

**Authors:** Orogun Daniel, Harriet Harris

**Affiliations:** 1 Department of Religion Studies, Faculty of Theology, University of Pretoria, Pretoria, South Africa; 2 Centre for Studies in Religion and Society, University of Victoria, Victoria, BC Canada; 3 University of Edinburgh, Edinburgh, UK

**Keywords:** Compassion, Science, Spiritual care, Caregivers, Healthcare, Methodology, Service delivery, Chaplain

## Abstract

Across the globe, spiritual care is offered by individuals, healthcare chaplains, and humanitarian, social and related spiritual groups on account of zeal, voluntary and paid services. Sometimes, services are provided without understanding the connectivity of compassion, spiritual care, and scientific protocols. There are instances where health professionals and managers disagree with spiritual caregivers or reject spiritual services because of poor service deliveries in conflict with healthcare protocols. Against this background, this article focuses on how spiritual care services can be provided scientifically to improve service delivery. It presents leading questions to link the scientific and compassionate approach to spiritual care. These include-What is science? What is compassion? What is spiritual care? What makes compassion and spiritual care scientific? Are there tenets of compassion in religions? How are compassion, science and spiritual care linked? What are the implications of the intersections for public health and safety? Hopefully, the provided answers may improve the service delivery performance of spiritual caregivers and their collaboration with healthcare professionals, social workers, and related groups.

## Introduction

A body of evidence from around the world demonstrates that providing appropriate and effective spiritual caregiving in the health sector is challenging for a variety of reasons, one of which is that some spiritual caregivers often adopt a poor approach to service delivery (Momeni et al., 2022, 575–580; Murgia et al., 2022; Rushton, 2014). This is the context in which this article discusses the intersections of spiritual care, science, and compassion to engender better services by spiritual caregivers in the healthcare sector.

It is critical to note that compassion can be traced to psychology which is a branch of science. Such psychological dimension is recognised and briefly alluded to here and there. But the context of compassion here is how it relates to the compassionate disposition of spiritual caregivers in healthcare. With the hindsight of the global perspective, four religions covering African, Western and Eastern global spaces are considered. These religions are African Traditional Religions, Buddhism, Christianity, and Islam. Their global demography, popularity, emphasis on spiritual caregiving, and availability of spiritual caregivers in healthcare are the reasons for choosing these four religions. However, the authors wish to state that choosing these four religions does not denigrate the import of other religions.

Moreover, this article does not suggest that compassionate spiritual care is a science, but that caregiving can be scientifically provided. In this sense, science speaks to the methodological algorithm of care that provides an acceptable culture to all stakeholders thereby engendering public health and safety. Thus, the objective is to show how a scientific approach can foster good spiritual care practices in the global healthcare ecosystem. It is to provide a methodological approach that allows spiritual caregivers to collaborate with healthcare professionals for effective multidisciplinary healthcare delivery. Against this background, the article clarifies the three concepts; compassion, science, and spiritual care, views them through the lenses of the four religions, and provides their intersections. Subsequently, the article discusses how spiritual care can be compassionate and scientific for public health and safety benefits.

### Understanding Science from the Perspective of Compassion

What is science? Various authors and dictionaries provide different perspectives on the definition of science. While the scope of this article will not permit all kinds of definitions, most resources consulted have a common denominator in the definition. Science Council (2009) refers to science as the pursuit and application of knowledge and understanding of the natural and social world following a systematic methodology based on evidence. Science is the methods to achieving such scientific knowledge and understanding including objective observation, gathering evidence, establishing general rules or conclusions from facts and examples, repetition, critical analysis, verification, and testing.

The Cambridge Dictionary (2019), submits that science is a careful study of the structure and behaviour of the physical world (nature) through measuring, watching (observation), experiments, developing theories and documenting results of activities. The most valuable information from the Cambridge Dictionary (2019) is that science is now a mass profession unlike being initially elitist, it is no longer separated from the life and concerns of ordinary people. This then suggests that knowledge of day-to-day life and recurring challenges of everyone is recognised in the world of science.

Further, Merriam-Webster (2009) defines science as a system of knowledge covering general truth or operations of general laws especially as obtained and tested through scientific method. This can be in any area like natural science, medical science or social science. The definition of Merriam-Webster (2009) is more fascinating because of the notion that science is a system or method reconciling practical ends for scientific laws and that it is scientific knowledge that distinguishes a system or operations from ignorance or misunderstanding. In other words, providing compassion scientifically means operating intentionally to avoid possible negative surprises engendered by ignorance or misunderstanding. The word scientific according to Merriam-Webster (2019), implies conducting an activity or practising something using thorough or systematic methods. For example, advertising can be done scientifically, baby care can be provided scientifically, and ditto compassion or spiritual care services.

Collins English Dictionary (2019) makes it simpler by defining science as any body of knowledge organised systemically. In other words, whether activities of religion, theology, sociology, psychology anthropology, as long as they are organised systematically and methodologically, they can be termed as scientific. Thus, to be scientific means to conduct observation, experiment, measurement, and formulation of laws to describe facts in general terms.

### Understanding Compassion

What is compassion? Goetz et al., (2010, 351) referenced the Oxford English Dictionary in defining compassion as the Latin word ‘compati’ meaning “to suffer with”, and it is interpreted as being motivated to help those suffering (cf. Lazarus, 1991, 289). In the opinion of Feldman and Kuyken, compassion is “an orientation of mind that recognises pain and the universality of pain in human experience and the capacity to meet that pain with kindness, empathy, equanimity and patience” (Feldman & Kuyken, 2011, 145). Berlant asserts that “it is an ethical calling. A part of being human among other humans in this world” (Berlant, 2004, 5).

Harris and Balaam further emphasise compassion as wisdom. In their words **“Compassion is attentiveness to the suffering of ourselves and others, with the wisdom and steps taken to relieve it. Compassion calls forth action but with the wisdom to know when, how and what is required”** (Harris & Balaam, 2021). The duo also distinguishes empathy from compassion. Empathy can easily lead to feeling overwhelmed because it gives caregivers insight into the feelings of others and can lead to getting swamped or stuck in the feelings of others, whereas compassion approached scientifically, provides wisdom (applied knowledge) on how to respond to patients’ or clients’ suffering without being overwhelmed (Harris & Balaam, 2021).

Additionally, Wheater (2023) understands compassion as culturally and historically contingent. Wheater calls it a contingent-wise action. This is connected to the definition of science because wisdom is the application of knowledge and most of the definitions provided in the previous section suggest that science is about the application of knowledge. Thus, compassion is about applying a system of knowledge in services of compassion. Both Wheater (2023) and Harris and Balaam (2021) could not agree more with an earlier assertion by the Dalai Lama (2005) that genuine compassion requires both wisdom and loving-kindness. For the Dalai Lama, it is wisdom to understand the nature of the suffering from which a caregiver wishes to free others, and it is an expression of loving-kindness to experience deep intimacy and empathy with other sentient beings ((2005), 49).

In consolidating existing definitions, Strauss et al. (2016, 15) list five elements of compassion. They include recognising suffering, understanding the universality of human suffering, feeling for the person suffering, tolerating uncomfortable feelings, and motivation to act to alleviate suffering. This addendum to definitions was based on operational meaning which demonstrates adequate psychometric properties. Gilbert (2010) also notes that compassion comprises six attributes which are sensitivity, sympathy, empathy, motivation or caring, distress tolerance and non-judgement. The first which is ‘Sensitivity’ speaks more to the definition of compassion as it involves being responsive to other people’s emotions and perceiving when they need help,

## What Makes Compassion Scientific?

Compassion may not necessarily be a science, but there can be scientific study and application of compassion by spiritual caregivers. A critical point made by Strauss et al., (2016, 19), has become a new compass for reviewing the definition of compassion. The five elements earlier suggested by Strauss et al., (2016, 15), seem to lack a scientific dimension. Yes, recognising suffering in others; understanding the common humanity of this suffering; feeling emotionally connected with the person who is suffering; tolerating difficult feelings that may arise; and being motivated to help the person, all play a significant role in understanding compassion. However, they all have notable psychometric weaknesses which may suggest serious limitations in the field. This is where the scientific approach to compassion now steps in.

A scientific approach to compassion may include objective observation, gathering evidence, measuring, experimenting, analysing, verifying, and establishing general rules, theories or conclusions from facts and examples. As an example, Strauss et al., (2016, 26) opine that without adequate measures, we cannot determine with any confidence, levels of compassion or whether interventions designed to enhance compassion are effective. They therefore called this scientific approach to compassion “empirical testing following good practice guidelines to identify items and test their psychometric properties” (Strauss et al., 2016, 26). This school of thought redefined compassion in a practical sense and set the model for caregivers. This is where compassion and science meet, ensuring an empirical approach to compassion.

### Spiritual Care in the Context of Compassion

What is spiritual care? Spiritual Care Australia (n.d.), defines it as how attention is paid to the spiritual dimensions of life and meeting the spiritual and emotional needs of individuals or groups. It includes presence, conversations, rituals, ceremonies, prayers, and the sharing of sacred texts and resources. A common denominator in most definitions of spiritual care is that spiritual care is an act of spirituality, provided as some form of professional social and healthcare services that engender coping with pain, trauma, loss and life transition (Scottish Government, 2023; Britt et al., 2021, 46–55; University of Maryland Medical Centre, 2024).

As a follow-up question-how can spiritual care find expression in compassion? Religiosity and spirituality were long associated with the epidemiology of compassion. In the research of Addiss, et al., 2022, 20), 13 spirituality and religion tests were conducted, and the result showed that 10 (76.9%) out of 13 tests imply an association with compassion. It then means for example almost 77% of caregivers who spend time praying for the sick, providing counselling to victims of addiction, or standing in support of the bereaved do so, not as a function of reward or fulfilling demands of routine tasks, but the expression of compassion to those affected. Correspondingly, (Armstrong, 2010) argues that compassion is popular across religions and spiritual traditions. Research reveals that it increases the quality of life of patients. For example, in the report of Balboni et al., (2011, 5383–91) patients with limited spiritual care experience more worries, anxiety, shortness of breath, and pain with higher chances of hospitalisation and death at intensive care units, instead of at home. Such a scenario puts patients and relatives in more emotional pain and financial costs.

Regardless of the value of spiritual care, Addiss, et al. (2022, 20) call to mind that religion can decrease the flow of compassion with issues like division, harsh dogma, and actions of injustice and discrimination, among others. Such assertions imply how spiritual care in healthcare ecosystems has become valuable. However, questions still abound on how well spiritual care is maximised. It is the opinion of this article that a more scientific approach to compassionate care by spiritual caregivers will yield more and better results for public health and safety. To further confirm how spiritual care connects with compassion, the next section looks at religious tenets on compassion as a form of spiritual care in selected world religions.

### Compassion in African Traditional Religion

According to Brown-Hinds (2015), in describing compassion in African tradition, compassion is like an ultimate manifestation of a spiritual nature. Baily II (2017) concurs that in African Tradition, both sympathy and compassion are God-made and share the same spiritual space*.* Although Baily II (2017) further claims that love shared in compassion services is unconditional and spiritual in Africa, but conditional in a European setting due to the involvement of emotion, such a claim may not necessarily be correct because much is also written about unconditional love via emotional expression in the European context (Fuller & Jasper 2021,1–280). Thus, in both African and Western settings, compassion can be unconditional. Overall, the unconditional and spiritual nature of compassion in various cultures especially in Africa, emphasises its importance in human society.

In the opinion of Brown-Hinds (2015), this spiritual nature expressed in Africans is by instinct communitarian, and it deals with healing and wholeness. Thus, the holistic nature of Africans’ view of healing informs the approach to compassion such that a caregiver or healer in African tradition considers that (1) the patient is part of a whole family of creation, and (2) the treatment protocol considers mental, social, and spiritual factors, rather than only the physical symptoms of an illness. As such, the holistic and communitarian position drawn from what Baily II (2017) calls “primordial interconnectedness”, helps spiritual caregivers to remember that all humans including patients share vital parts in and of the same spiritual space; and remain in constant communication. This idea forms not only the beginning of African tradition on earth but also the basic concept of compassion.

Additionally, Brown-Hinds (2015), seeks to suggest that compassion stems first from a belief system in African tradition. Mbiti, an East African scholar was spot-on in this regard when he concludes that “What people do is motivated by what they believe, and what they believe springs from what they do and experience. So then, belief and action in African traditional society cannot be separated: they belong to a single whole” (Mbiti, 1970, 5). Worthy of note is how Nussbaum from the Southern African perspective further connects compassion to Ubuntu. In his words, it is “the capacity in African culture to express compassion, reciprocity, dignity, harmony and humanity in the interests of building and maintaining community” (Louw, 2020, 2–4; Nussbaum, 2009, 100; Van Niekerk, 1994, 2). Thus, compassion in Africa is not a wish of individuals but a community culture.

Brown-Hinds (2015) further expresses that the application of compassion in African tradition follows a few protocols. It starts with “Felt” compassion which in the Akan language of Ghana in West Africa is called *Ayemhyehye*. This first level of compassion begins with a burning, churning, or gnawing sensation within the stomach produced when a compassion service giver faces human suffering in connection with pain, ailment, loss, lack, obstruction, or desolation (cf. Harris 2021, 119). In linguistics exposition, Sakyi and Nuyts (2019, 109), use the phrase “With compassion, pray” to express the active element that follows *Ayemhyehye* or the sensation of compassion when confronted with someone in need. Both the sensation and linguistic interpretation suggest that compassion is both instinctive and spiritual. As the instinctive and spiritual grow, the prospective caregiver develops *Ahummobor*; a state of being which represents the mindset of being open to another person’s unbearable pain and suffering and developing a strong inclination to relieve such misery. Afterwards, the third protocol called *Abadai* steps in. It is interpreted as a beneficence act (care and gift-giving) offered by the caregiver to reduce or stop, or in other cases reverse the condition either through traditional or spiritual therapy.

These traditional actions delineated above-*Ayemhyehye*, *Ahummobor* and *Abadai* may pass for the words of Mbonu Ojike in Opoku (1978, 3) when discussing African social, traditional, and spiritual practices in the community. In his words “But if religion means doing rather than talking, then Africa has a religion” (Opoku, 1978, 3). Further, Brown-Hinds (2015) notes that such compassion protocols need wisdom that springs from empathy, observation, and caring. Again, this assertion suggests a scientific and systematic application of knowledge (wisdom) in compassion and spiritual care services. It suggests that Africa has an indigenous knowledge system to provide compassionate spiritual care services scientifically. Overall, the assertions of Sakyi and Nuyts (2019, 109), Brown-Hinds (2015), Opoku (1978, 3) and Mbiti (1970, 5) show how the act of compassion in African communitarian society is firstly spiritual and that such spiritual understanding needs compassionate and scientific protocols to ensure holistic spiritual care services.

### Compassion in the Christian Tradition

Throughout the Christian literature, evidence abounds that compassion is one of the tenets of Christian practice. Using David as a case study in the Old Testament, Jonathan in a lifesaving situation expresses his need for compassion (a synonymous word for kindness) from David (1 Samuel 20:14). David promises to show the same to Hanun the son of Nahash, in reciprocity to the father’s kindness (1 Chronicles 19:2). As evident in 2 Samuel 9:1–7, it appears that reciprocity of compassion became David’s culture as he promised Mephibosheth, the son of Jonathan. David’s words in Psalm 31:21, seek to suggest that he is thankful for God’s expression of compassion when he was in a city under siege. The scenarios above show how Christian belief suggests that compassion, is transgenerational and can be inferred as cultural or civilisation in the days of David, ditto the culture of African Tradition.

In the New Testament, the Beatitudes in Matthew 5: 3–12 represent a follow-up of David’s thoughts on compassion. More specifically, in Matthew 5:7 Jesus asserts that “Blessed are the merciful, for they shall receive mercy.” The scripture suggests that one who shows mercy gets mercy, ditto one who supplies compassion. More so, Julia (2020) agrees no less that mercy is the fruit of compassion in the Christian perspective. Further, using Jesus as the New Testament case study, six scriptures reveal his love through his compassion in the miracles he performed. They include compassion for the dead in Luke 7:13; the hungry in Matthew 14:14 and 15:32; the sick in Matthew 20:34 and Mark 1:41 and the demon-possessed in Mark 5:19. Paul in his Epistles also sustains the culture of compassion in his teachings (See Galatians 15:13; Colossians 3:12; cf. Sorren et al., 2022). Overall, the love of Christ is expressed in compassion. Pembroke was then right when he concluded that compassion service in the Christian faith is founded on agape (Pembroke 2004, 143–44). In this context, agape means providing “sacrificial love that intentionally desires another’s highest good” (Roat, 2019), and not being unconditionally compassionate to oneself alone but focusing more on others around with care (Fleming, 2024).

The culture of compassion did not end with bible scenarios, it inspires spiritual care providers to do the same. Christian leaders over the years, including Aquinas who asserts that compassion is integral to the virtue of mercy, see receptivity and disposability to compassion as critical for Christian practice. Aquinas (1225–1274) defined the virtue of “mercy” in his great Summa Theologiae (ST II-II. 30.1) as “the compassion in our hearts for another person’s misery, a compassion which drives us to do what we can to help him.” (Cates, 1997, 229). For St. Thomas Aquinas, this virtue has two aspects: “affective” mercy and “effective” mercy. (Lobis, 2015; Cates, 2009; Pembroke, 2004, 129; Cates, 1997, 229). Thus, in contemporary times where some medical educators dislike teaching or heralding compassion (Balaam and Harris, 2021:369–376), it is expected that healthcare chaplains, social workers, healthcare professionals and managers need to develop a culture of accommodating and adopting compassion as an ethical and frontline principle in service delivery (Van der Cingel, 2009, 124–136).

### Compassion in Islamic Tradition

The Islamic tenets of compassion are rooted in the 5 pillars of Islam. According to Islamic laws, Zakat as one of the 5 pillars is compulsory for every sane adult Muslim who owns wealth over a certain amount-known as the *Nisab*. They must donate 2.5% of their entire income for humanitarian services. These may include building mosques, providing water, hospitals, and schools. Zakat is done as a religious duty and act of charity to invoke Allah’s blessings (Canby, 2019; The Editors of Encyclopaedia Britannica, 2019). While Islam encourages Muslims to pursue economic and social profits, it reminds them not to be selfish but to care for others by sharing what they have. In some countries, the government takes responsibility for the collection and sharing of Zakat (The Pluralism Project Harvard University, 2020).

The narrative above suggests that compassion for others in the community is fundamental in Islamic tenets. Though it can be argued that some state-controlled Zakat collection, makes such humanitarian acts not compassion-inclined but compulsive exercise. However, the affective element whereby givers are moved to comply with the Zakat law because of the needs of the less privileged or others suggests that voluntary compassion cannot be ruled out of the equation. This argument is supported in the next two paragraphs below.

Either in Islamic medicine or otherwise, compassion is both a religious obligation and voluntary. According to Ibn ‘Umar, Prophet Mohammed stated that “The Muslim is the brother of his fellow Muslim; he does not wrong him or let him down. The one who meets the needs of his brother, Allah will meet his needs. Whoever relieves a Muslim of distress, Allah will relieve him of distress on the day of resurrection” (The reward of taking care of one who is sick-Islam Question & Answer. 2012). Furthermore, according to the narrations of al-Bukhaari (2442) and Muslim (2580), anyone who stays with the one who is sick and takes care of him and looks after him has done good by serving him and caring for him. Allah loves *Al-Muhsinoon* interpreted as the good-doers (Haider, 2017).

While it is an Islamic etiquette to show compassion by visiting and supporting a sick Muslim, it is much interesting that Prophet Mohammed encouraged that non-Muslims should not be excluded. This suggests that showing compassion sets aside biases and has the power to unite people of different religious orientations (Sun 2017). The first visit to the sick is *Sunnah* meaning a customary or obligatory act expected of a Muslim, while the subsequent visit is voluntary. The Prophet equally attached a reward of *al-Jannah* (Paradise) to those who show care to the sick (Sun 2017; Demystifying Al Jannah, n.d.)

### Compassion in Buddhism

Like other religions, compassion in Buddhism is popular, especially in psychology literature. According to the Dalai Lama (1995), compassion is openness to the suffering of others with a commitment to relieve it. Beyond emotional response to the sufferings of others, it is a response founded on reason and wisdom; an ethical framework which advocates selfless intention of supporting others to free them from suffering (Strauss et al., 2016, 16–17; Lama & Chan, 2014). In other words, in Buddhist conceptualisation, compassion goes beyond affective and behavioural elements but also consists of cognitive components as far as it involves being able to imagine and reason about a person’s experiences (Strauss et al., 2016, 17).

According to the Dalai Lama in Lonczak (2019), religion has a responsibility to fill the gap created by Western civilisations which places great importance on filling the human ‘brain’ with knowledge, while ignoring filling the human ‘heart’ with compassion. In the Dalai Lama’s opinion, filling this gap is what the real role of religion is (Lonczak, 2019). Lown et al. (2011) corroborate the fact that there is a gap with the notion that only 53% of those hospitalised get compassionate care. However, this assertion may be debatable because it does not specify whether it is the compassionate care that is insufficient or only 53% of the patients perceived they have received care. While it is important to underscore that the claim is debatable, the focus of this paper remains on the authors’ suggestion that compassionate care by spiritual caregivers in healthcare needs to increase or improve. On this premise, one may agree with these authors’ submission that there is increased attention on compassionate care among researchers, especially in nursing, but compassion training remains a grey area needing more attention and improvement (Lown et al., 2011, 1772–1778).

Overall, Buddhism shows a strong tradition and faith in commitment to compassion especially in the healthcare sector. The fact that Buddhism calls the attention of religion to the humungous gap in training and practice of compassion, represents its strong tenets on the subject.

Concomitantly, the gap delineated above is also attested to by other assertions. Pembroke (2004, 140–41) asserts the distance between healthcare professionals and patients caused by medical technology alongside extreme time pressure. These challenges often lead to squeezing out compassion in medical practice. This could be why Van Der Cingel (2009) concludes that medical care too often lacks a human face. Van Der Cingel (2009, 124–136), equally contends that a lack of compassion in what he calls clinical empathy can potentially increase patients’ suffering and the risk of diagnostic and management errors (cf. Halpern, 2003, 670–74). All these assertions align with Buddhism to make compassion services part of nursing philosophy. Beyond religious groups, Buddhism is encouraging the spread of compassion-focused therapy among healthcare professionals and related organisations (Leaviss et al., 2015, 927–945). This is a show of commitment to and the popularity of compassion in Buddhism.

### Intersections of Compassion, Science, and Spiritual Care

Earlier, under the scientific nature of compassion, it was delineated that compassion can be presented scientifically, ditto spiritual care. Under the section on spiritual care in the context of compassion, there exists a strong link between spiritual care services and compassion. Throughout the religious tenets presented earlier, the selected religions advocate the obligation of compassion as a part of religious duties.

Islam does not only make compassion a religious obligation but promises access to *al-Jannah* as a reward for the acts of compassion*.* In African traditional religion, compassion is both instinctive and spiritual. Therefore, if compassion is spiritual, there is a link between compassion and spiritual care. Just as the African spiritual tradition is built on “primordial interconnectedness and healing and wholeness, salvation in Christianity seeks connectedness of humanity and divinity. It speaks to the connection of spirit, soul, and body (wholeness). It speaks of compassion and the love of God leading to the sacrifices of the compassionate Jesus.

Likewise, psychology as a field of science may regard compassionate expression as some form of connectedness between a giver and a receiver. This assertion is made on the account that “Compassion operates through evolved psychological and physiological mechanisms that underpin our mammalian caregiving motives and behaviour” (DeMarco, 2023). Thus, science, compassion, and spiritual care, meet in the place of the connectedness of the caregiver and the receiver. Moreover, Buddhism infers that spiritual care bridges a major gap; medical professionals provide services with their brains while compassionate caregivers support patients with their hearts. Besides, some medical practitioners also apply their technical medical knowledge with compassion. Therefore, when the heart and the brain (science and spirituality) function in the healthcare ecosystem for the same purpose, a link is inevitable. Such a link provides robust and holistic care.

With the background of the intersections, a leading question is whether spiritual caregivers can be more scientific in providing compassionate spiritual care. Response to such questions will be affirmative if spiritual caregivers are willing to embrace this article’s later submission in the next section. But in the meantime, it is important to mention that although spirituality cannot be measured, the by-products which may include the responses, feelings, and testimonies of patients, can be observed, analysed, verified, and serve as evidence upon which the scientific nature of spiritual care can be tested as reliable. Consequent to the results of the tests, robust rules of engagement can be developed for spiritual care in healthcare.

## Steps to a more Scientific Approach in Caregiving

It is important to recall that this article aims to see how caregiving can be more scientific (methodological) to allow ideal and acceptable practice for all stakeholders in the health ecosystem. With that being said-what methodological protocol can be adopted by spiritual caregivers? Five points are provided below. But one must add that it is not the purpose of this article to deskill excellent spiritual caregivers whose services are acceptable and highly appreciated by healthcare professionals and managers. The wish here is that these captures will improve their existing capabilities. Below are the points to guide spiritual caregivers.

- ***The first step is knowing and understanding the nature of scientific caregiving:***
  Under the section ‘understanding science from the perspective of compassion’, having the knowledge and understanding of the natural and social world following a systematic methodology based on evidence, is the foundation of becoming scientific in caregiving. A caregiver ought to seek knowledge of the structure and behaviour of the physical world (nature) and understand the same. There will be nothing to base spiritual care services upon without knowledge. Knowledge of human nature, behaviour, and reactions to pain, troubles, and trauma among others is important.
  Besides knowledge of religious texts, having a broad spectrum of knowledge covering human, medical and social science is imperative for a scientific approach to caregiving. This is why some spiritual caregivers in climes like America and Canada prescribe Clinical Pastoral Education and Clinical Psychospiritual Education for spiritual caregivers in hospital environments (See Canadian Association for Spiritual Care (n.d); Association for Clinical Pastoral Education 2020).
  Furthermore, to seek knowledge infers that to become a scientific caregiver requires an understanding that spiritual care is educational. It means compassionate caregiving is not all about being spiritual, it can be learnt and improved. For example, a spiritual caregiver needs to be altruistic, non-judgmental, practice gratitude, and be kind to self and others. These requirements are not instinctive, nor can spirituality replace them. It is therefore important for a caregiver to know and understand that learning the basics of compassion is a requirement in spiritual care.
- ***The second step is to understand and apply the methodological approach:***
  Based on the literature discussed so far, some usable methods of a scientific approach to caregiving may include but are not limited to the following.
  1. Objective observation of patients’ responses, feelings, and testimonies or the activities of an experienced caregiver.
  2. Gathering several of the observed cases.
  3. Repeating the process of observation in closely related or similar cases.
  4. Compiling as many cases as possible as evidence.
  5. Allowing verification of the compiled pieces of evidence.
  6. Critically analysing the compiled evidence
  7. And then drafting theories, and general rules including ethical protocols or making conclusions from facts, examples and evidence gathered.
  8. Ultimately, allowing the heart (compassion), head (knowledge) and spiritual energies to process points 1–7 above and thereby guide how spiritual care services can be improved going forward.
- ***The Third step is to seek wisdom to apply the knowledge and methods:***
  As delineated earlier, Christianity, Islam and Buddhism suggest that spirituality has a role in caregiving services. Further, the linguistic interpretation of compassion (*Ayemhyehye*) under the section of Compassion in African Traditional religion suggests that the expression of compassion is both instinctive and spiritual (Sakyi & Nuyts, 2019, 109). However, it is important to note that the scientific nature of caregiving makes it not only instinctive and spiritual, but also wisdom-related. Wisdom is about the application of knowledge.
  Feldman and Kuyken (2011, 145) assert that compassionate caregiving requires the orientation of the mind to recognise pain in human experience and develop the capacity to meet that pain with kindness. Harris and Balaam (2021) believe that compassionate caregiving requires **wisdom to know when, how and what is required. Wisdom helps the compassionate caregiver provide support for the patients without being overwhelmed.** Wheater (2023)** calls it** a contingent-wise action. The Dalai Lama thinks that wisdom helps to understand the nature of the suffering from which a caregiver wishes to free others ((2005), 49). For example, it takes wisdom for a caregiver to understand the closeness and context within which services should be provided. Caring relationships and receptivity may differ and caring for others is a costly task, and therefore wisdom is required to know when, how and the wisdom required to approach situations depending on the context, closeness, or receptivity (cf. Gilbert, 2019, 3).
- ***The fourth step is to have a broader and collaborative mindset:***
  A caregiver does not consider the spiritual aspect of healthcare as a stand-alone exercise, but as part of a holistic care system called the Extended Biopsychosocial model (Hefti, 2011). Another instrument for a broader and collaborative approach is Sulmasy’s Biopsychosocial-spiritual framework; a model that promotes a genuinely holistic health care where the totality of the patient’s relational existence-physical, psychological, social, and spiritual are under consideration during spiritual care (Sulmasy, 2002, 24–33). Thus, a compassionate spiritual caregiver needs to have a broader lens of approach; noting the need to function alongside healthcare professionals, social workers, and psychologists, among others. Where possible, knowing and understanding the language and rules of other professionals and the operating environment is critical for a holistic approach. For example, it would be wrong for a caregiver to insist on time to pray for a patient when the doctors need time to observe the patients’ reactions or responses to specific drugs, injections, or procedures. Spiritual caregivers must understand that healing takes specific processes and requires protocols and methodologies and therefore must be guided accordingly. Even in end-of-life care, laid down rules must be religiously observed.
- **The fifth step to becoming scientific requires some levels of cognitive competencies through pieces of training:**
  Gilbert (2019, 3) suggests that human compassion services require a specific set of cognitive competencies. Some of the competencies have continued to evolve over the last 2 million years (Dunbar, 2016; Suddendorf, 2018). They include a variety of complex reasoning capabilities that allow several forms of self-awareness, symbolic and systemic thinking, mentalising, reflection on the past, and behaviourally contingent predictions of the future called ‘mental time travel’ [if I do that, then this is likely to be the outcome] (Suddendorf, 2018). All these competencies are imperative for a scientific approach to compassionate spiritual care. These are not evidence-based training, but professionally validating training that comes with competency improvement in spiritual care services. Some parts of the Western hemisphere have seen recent developments in this area with pieces of training around psychospiritual, psychological, and social processes (Kirby et al., 2017). Taking a cue from the West may be valuable for other regions like Africa as different sources show that cognitive competencies can be developed from psychospiritual, neurological, psychological and social pieces of training (Gupta et al., 2019; Henrich et al., 2023).

Other important areas of competencies training needed are compassionate values and morals, courage, and dedication. These competencies are not automatic, they are deliberately chosen and worked at with a deepening of understanding over time. Although a heart of love for humanity is the foundation for compassionate spiritual care, it does not need just love, it needs “commitment from a perspective of cognitive competency” (Gilbert, 2019, 10).

### Public Health Benefits

The discussion about the implications of a scientific approach to spiritual care in public health includes public safety. This is because public safety influences community health in the areas of physical and mental well-being, social behaviours, and the emotional safety of patients. Thus, a safe community is a healthy community. The emergence of COVID-19 has also helped organisations and government agencies to realise the need to blend public health and safety. Hence, there are emerging policies on how to include public safety in healthcare protocols (University 2021; Blurring the Line between Public Health & Public Safety-Bill of Health, 2021; ‌Public Health and Public Safety, n.d.). On this account, the public health implications of a scientific approach to spiritual care will be juxtaposed with public safety in this section.

The context within which spiritual care can be scientifically provided requires the knowledge of operations, protocols of administering spiritual care, theories, rules, observations, analysis, and verifications. These pieces of knowledge will guarantee quality spiritual care and strengthen patients’ healthcare and safety. The following are some positive implications of administering spiritual care under some scientific approaches discussed in the previous section.

1. *** Cooperative behaviour and checks and balances:***
  Having a scientific approach provides the opportunity for spiritual caregivers to improve their knowledge system and principles of medical ethics which guarantees standard healthcare and the safety of patients and caregivers. It allows champions of public health, health systems, health ministries and private practitioners to easily accommodate and collaborate with spiritual caregivers. This is what Van de Cingel (2011) calls “Cooperative behaviour”.
  Further, embracing a scientific approach may reduce the conflict between spiritual caregivers and healthcare professionals. It can check the excesses of spiritual activities of caregivers which may jeopardise the healing, recovery and safety of patients, and guarantee respect for human dignity in a healthcare environment (Department of Health & Social Care, 2012; Grounds et al., 2010). Moreover, compassionate caregiving may motivate the desire to comply with whatever rules are set to ensure holistic healing and recovery of patients. A caregiver cannot be compassionate about patients’ well-being and still disregard the rules of engagement for their healing and recovery. This is why compassionate and scientific approaches to spiritual care are imperative for public health and safety.
2. ***Job satisfaction, organisation commitment, and the flourishing of Community:***
  A methodological approach to spiritual care which includes building an appropriate learning system, can create a more flourishing community. The more scientific the caregiving, the better the technical know-how is developed. This then leads to service satisfaction, a sense of emotional vigour and reduced burnout among compassionate spiritual caregivers (Harris & Balaam 2021; Eldor & Shoshani, 2016). When a caregiver comes to the table with compassion, commitment is almost a default element, and such commitment further leads to a desire to be methodological and compliant with all rules leading to holistic healing and patient safety. Once these positive elements dominate the caregiving environment, healthcare is improved and the community flourishes.
3. ***Public confidence in caregivers’ services and reduction of risk of harmful experiences:***
  The more scientific the process of caregiving becomes, the better the results and the flourishing of health in the community. This will engender patients’ satisfaction, lower distress and the community’s confidence in spiritual care services (Lelorian et al., 2012). The more scientific the services are, the less the risk of harmful experiences. Likewise, the less the risk, the safer the patients and caregivers. Reports have shown that where compassionate and spiritual services are increasingly adopted, the lesser the harm and the higher the levels of well-being and survival rates (Ironson et al., 2016; Zessin et al., 2015).

## Research Limitation

This article presents the scientific nature of spiritual care from a more methodological perspective. It discusses usable methods to foster effective and collaborative service delivery performance of compassionate spiritual caregivers while working alongside healthcare professionals and managers. However, the article is not without limitations. A notable limitation is that it did not provide a psychological approach to spiritual care services. This gap can be explored in further research. Therefore, the article provokes thoughts on further discussion of the psychospiritual approach to spiritual care service delivery in a healthcare environment.

## Conclusion

This article answered questions raised in the abstract concerning the intersections of compassion, science, and spiritual care in three areas. Firstly, it showed the connectedness of compassionate caregivers and receivers. Secondly, it revealed the connectedness of religious tenets (spiritual) and compassionate services. Thirdly, it showed the connectedness of science (methodology) and spiritual care. Another question was whether care could be provided scientifically. In answering the question, 5 steps on how spiritual care can be scientifically provided were presented to engender best practices and improve public health and safety. Finally, the question of public health benefits was addressed by the provision of 3 areas where a scientific approach to spiritual care engenders positive implications. Overall, this paper opined that a scientific approach to spiritual care can promote collaborations, improved services, public health, and safety.
